# Young Women as Change Agents in Sports and Physical Activities in the Punjab (Southern) Province of Pakistan

**DOI:** 10.3389/fpsyg.2022.857189

**Published:** 2022-06-17

**Authors:** Rizwan Ahmed Laar, Shahnaz Perveen, Muhamad Azeem Ashraf

**Affiliations:** ^1^College of Physical Education, Hubei Normal University, Huangshi, China; ^2^Department of Education, Government Sadiq College Women University, Bahawalpur, Pakistan; ^3^Education Science Research Institute, Hunan University, Changsha, China

**Keywords:** physical activities, women’s empowerment, gender stereotype, feminism theory, the international implication, change agents

## Abstract

Women’s empowerment is a concept describing the promotion of women doing things independently and in their own interests, being more conducive to their future and physical and mental development; this includes participation in different outdoor activities, including sports. This qualitative study presents data collected from 18 young female students at sports and physical education universities in Southern Punjab (SP) in Pakistan, selected using a snowball sampling technique. The current study explores their gendered and lived experiences of playing sports and engaging in physical activities in patriarchal systems by emphasizing the concept of women’s empowerment in the context of feminism in sports theory. The findings suggest that the participants faced typical gender stereotypes in their families and communities, which position sports and physical activities as being not feminine. The chances for women to participate in sports and physical activities decrease as they grow up. However, the participants used a range of strategies to advance their interests and academic careers in sports and physical activities by resisting and incorporating dominant discourses of women’s participation in sports and physical activities, which also has implications in the internal and external policy domains at the local and national levels. The participants displayed great resilience and optimism, empowering them to enter the male-dominated domains, and thus we labeled them as change agents.

## Introduction

Women’s empowerment is a concept that recognizes women’s influence over subjective choices in their own lives; thus, it has a resilient basis in human rights (Hargreaves, 1994). Women’s empowerment builds an environment within which women can make deliberate decisions and premeditated life judgments in certain settings. Women form nearly half of the world’s population, and women’s empowerment is thus considered a crucial element in attaining the maximum level of desired development (Klasen, 2006; Upadhyay et al., 2014). The concept of empowerment through sports and physical activity has become a tool for uniting women in their struggle against patriarchal customs regarding women’s choices regarding their lives and bodies (Bradshaw, 2002). Women’s empowerment through physical activity is an important element for a healthy lifestyle (see Peters et al., 2008; Cronin et al., 2019) in both developed and developing countries. The existing discourses have shaped the ordinary perception of physical activity and directed the development of global and local policies for women’s participation in sports and physical activities.

Women in Pakistan legally enjoy equal rights with respect to their name, voting, education, social and political contribution, and participation in physical activity. However, sports and physical activities are still excluded from the debates surrounding women’s empowerment in Pakistan (see the National Institute of Population Studies, 2019). The Islamic Holy Book (Quran) and the traditions of the Prophet Mohammed (Hadiths) support physical activity as an important part of the development of human health. Good health and fitness are described as a boundless consecration in Islam. Islam stresses the growth and preservation of bodily and mystical powers irrespective of gender differences (Bakhtiar and Majumdar, 2007). Islam openly motivates Muslims to guide their offspring to learn various physical activities to make them physically strong and active. The Prophet Muhammad (PBUH) said that Muslims should guide their children in learning the arts of horse riding, throwing arrows, and swimming (Muslim and Muslim, 2007). Although physical activity is encouraged for all Muslims, including women, women’s participation in sport and physical activity is perceived to be restricted through some religious and cultural rules (Laar et al., 2019a). Women in Pakistan, similar to those in other Muslim and regional countries, have stated that physical activity is incompatible with religion, with the modest dress requirements and lack of segregated facilities (Edim and Saba, 2014; Laar et al., 2019a), cultural norms arranged by families and communities, personal interest, and economic aspects (Laar et al., 2019a) being cited as major factors influencing their participation in sports. The belief of whether performing physical activity lies within the framework of Islam is a major consideration. Islam is not against women participation in sports and physical activities, as there is no provision in the Quran that prevents women from partaking in sports activities as long as women fulfill the requirements in terms of modesty (Hargreaves, 2007; Dagkas et al., 2011). Similarly, Benn et al. (2010) highlight the same matter, whereby the provision of equal opportunities to girls in sports needs to be harmonized with the Islamic framework in terms of wearing modest dress. Another cultural norm that indicate is the fear of de-feminization due to participation in sports activities. Women are strongly influenced by the practices of cultural preservation nurtured through socialization, and therefore, women avoid being involved in sports and physical activities. This could be a result of beliefs among societies that women will become physically stronger and perhaps take on masculine characteristics in society (Daiman, 1994). The de-feminization of women as a result of entering male-dominated public spheres may be perceived as a challenge to social norms that try to limit the role of women. It is impossible for women in Pakistan to participate in national and international events if they do not learn and develop the skills required to play their sports of interest. Thus, the exclusion of women from public domains may decrease their ability to select a career as an athlete, violating the human right of women to participate in physical activity at national and international sports competitions.

This study is a part of our project examining women’s empowerment in Pakistan on the basis of their lived experiences, and focuses on how young women in Pakistan are empowering and disempowering themselves with respect to physical activities. These young women in Islam studied physical education and sports at one public university located in the Southern Punjab (SP) region of Pakistan. We refer to these women as change agents, as most of these young women were the first in their families and one of few in their communities to participate in physical activities and to leave their homes for sporting activities. These women, as change agents, are founding a path for other women in their families and communities and creating opposition to the cultural and religious norms present in their families and communities. This study discusses the need to analyze the issues aiming to empower women in their specific cultural and social contexts, intertwined with religion and culture, in light of feminism in sports theory. This confronts the linear narrative of empowerment through physical activities by signifying the ways in which physical activities can construct new gender relations that can immediately empower and constrain women in different contexts. Furthermore, our study problematizes these narratives by underlining how certain relations and factors shape young women’s actions and relationships together with their attentions, inspirations, and goals to live a life of their own choice, which can have international implications in the context of women participation in sports.

## Research Design

This study emerged from our project on young women’s empowerment in Pakistan, focusing on women’s perceptions about physical activities for a healthy lifestyle and their lived experiences of participation in physical activities. Similar to other studies on women’s experiences in physical activities in Pakistan (Fazal et al., 2019; Laar et al., 2019b), we adopted qualitative, exploratory, and inductive methods to collect the data. The collected data were analyzed using a thematic analysis approach. First, codes were created that correspond with main themes and, after refinement of the themes, a report was produced (see Corbin and Strauss, 2014; Laar et al., 2021). To assess the situation of sports participation of female Pakistani students, qualitative results are provided in the current study. Qualitative studies are the best way of understanding and explaining an interviewee’s statements about his or her specific past attitudes and experiences (Rosenthal, 2004). Similar to other studies on women’s experience in sports (Mirsafian et al., 2014; Lenneis and Pfister, 2017) and in Pakistani society (Fazal et al., 2019), we conducted semi-structured and informant-style interviews. The participants were interviewed in Panjabi (the local language) and the interviews were translated into English. The participants were enrolled in a 2-year master’s program—M.Sc. Physical Education & Sports Sciences (further discussed below in detail). All of the program faculty were male, reflecting the gender inequality in Pakistan’s sports and higher education settings. However, the research mainly focused on the young women’s experiences of playing sports and participating in physical activities before enrolling in this program, which means that this research does not include the young women’s experiences of learning and playing sports at university.

### Background Information of Participants

The participants were between the ages of 20 and 25 and were from Bahawalpur, Multan, Bahawalnagar and Rahim Yar Khan, located in the SP region (Table 1). Most districts in the SP region have minimal empowerment of women, with a large disparity between urban and rural populations in the selected dimensions of education, independence, and housing services (Khalid et al., 2020). One or both of each participant’s parents was educated and had attended university to a bachelor’s or master’s level, and this was an important factor in encouraging them to enter higher education and choose a male-dominated major. Most of these young women selected physical education mainly because of the support they received from their parents. However, some of the young women were subjected to great pressure and faced a large number of obstacles in getting their parents (mostly their fathers) to agree with their choice of major in higher education. Physical education had previously been considered to be and described as a male-only profession for most of these young women, until they chose to change this patriarchal structure.

**TABLE 1:** Demographic information of the participants.

Participant (Anonymized)	Age	Hometown	Program level	Noteworthy asset
P*1	25	Bahawalpur, SP	2nd semester	First-generation woman in higher education; first woman from family in physical education
**P2**	24	Lodhran, SP	4th semester	First woman from family in physical education; chose major against some family members’ will
**P3**	22	Bahawalpur, SP	2nd semester	First woman from family in physical education
**P4**	20	Bahawalpur, SP	2nd semester	Travels alone to the playgrounds for sport(s) despite brother being unwilling
**P5**	21	Bahawalpur, SP	2nd semester	Plays sports for own interest, despite family not liking her playing sports
**P6**	22	Bahawalnagar, SP	4th semester	First woman from family in physical education
**P7**	20	Bahawalpur, SP	4th semester	First woman from family in physical education
**P8**	23	Bahawalpur, SP	2nd semester	First woman from family in physical education; arranged her own fee from part-time tutoring
**P9**	20	Bahawalnagar, SP	2nd semester	First woman from family in physical education
**P10**	21	Bahawalpur, SP	3rd semester	Lived abroad; first woman from family in physical education
**P11**	20	Rahim Yar Khan, SP	2nd semester	First woman from family in physical education
**P12**	20	Rahim Yar Khan, SP	1st semester	First woman from family in physical education
**P13**	20	Multan, SP	1st semester	First woman from family in physical education; Selected physical education even though parents did not like
**P14**	22	Bahawalnagar, SP	1st semester	First woman from family in physical education
**P15**	22	Bahawalnagar, SP	2nd semester	First woman from family in physical education
**P16**	21	Bahawalpur, SP	2nd semester	First woman from family in physical education; selected this major against elder brother’s will
**P17**	22	Bahawalnagar, SP	2nd semester	First woman from family in physical education
**P18**	22	Bahawalpur, SP	3rd semester	First woman from family in physical education

P*, participant.

### Sampling

Eighteen young female students from one public sector university located in the Southern Punjab (SP) (the largest province of Pakistan) region of Pakistan participated in this study. These young women were enrolled in the Master’s program in physical education, and we denoted them as change agents in contemporary Pakistani society, because many of them were the first among their families and societies to select physical education as their major. The authors approached the respondents by means of an official procedure, first contacting the heads of departments, then reaching out to the participants with the help of snowball sampling (Laar et al., 2019b,^2020^). All participants gave their informed consent in written form prior to their participation in the study. The study was conducted in accordance with the Declaration of Helsinki, and the protocol was approved by the Ethics Committee of the College of Physical Education, Hubei Normal University. We fully complied with all relevant ethical considerations. We elicited the narrative of their personal experiences of empowerment before and after enrolling in the program, explored what factors supported and constrained their choice, what aspects of society these young women considered had empowered them to enter into a male-dominated arena, and how these women were setting examples for other young women in a patriarchal society. With respect to the local cultural and religious values, the second author, a female academic and feminist working on several projects for training young women, contacted the participants and conducted interviews. Throughout this study, ethical considerations were followed with respect to the participants’ privacy and the confidentiality of the research data. All participants were informed about the aims of the study before the interview, and that the anonymity of their identity, including their affiliation, would be assured. The participants were provided with a further explanation about the scope of this study at the time of the interviews. Participants signed a written informed consent form to verify their voluntary participation in this study. The contents of the questions were sent to the participants ahead of the interviews, but were explained again before the formal setting of the interview.

### Data Analysis

For data analysis, we employed three-stage systematic data analysis (Saldaña, 2012) to summarize the responses of the young women who participated in this study. By following the guidelines of Saldaña (2012) for systematic data analysis, in this approach, we first developed a detailed familiarity with the data through repeated readings of each interview transcript. In the first stage, we divided all transcribed data into open codes after examining the similarities and differences among the reflections provided by the young women. In the second stage, these open codes were further divided into meaningful categories to distinguish their relationships and properties. In the final stage, we developed particular themes in which the initial codes and meaningful categories were unified around essential categories. This approach to developing the specific themes over three stages helped us stay close to the participants’ meanings, as the codes are short symbolic indications that capture the spirit of the transcriptions (Saldaña, 2012). This strategy facilitated a better understanding of the young women’s perceptions and priorities, and it enabled us to remain open to the amazing aspects of their lived experiences.

## Findings

The choice to play sports for young women in this study and to select it as a learning major at university involved shared concerns related to their interest in sports. All participants were the first women among their families to select sports and physical education as learning majors at university. In contrast, most of these young women were first-generation students in higher education. The responses from the participants produced several common themes that had enabled them to enter into the male-dominated spaces of sports and physical education. The lived experiences of these young women before and after entering these patriarchal spaces could yield amazing outcomes for next-generation women in their own families and their communities.

### Change Agents: Incorporation and Resistance

This section emerged from the denials and challenges the participants confronted when attempting to achieve their desire to play sports. Different practices of resistance and adaptation helped the participants to position themselves by drawing on dominant social discourses and rejecting the social pressure presented by families and communities. In this regard, P1 (Table 1) shared her story, as follows:

*My aunt advises me to stop playing sport(s), as this field has many issues for girl players. But in spite of her advice, I cannot leave this field due to my immense interest in it. My father has also asked me a few times to leave this field. But I did not do this.*

P5 said

*My family allowed it at Bachelor’s level due to there being a female institution. But after* the *completion of my Bachelor’s studies, my father would not permit me to continue these sport(s). My father advised me earlier, “my dear! These sport(s) are not your future. You should focus on your academic studies only.” So, I agreed with my father and almost left sports. Moreover, our society also does not like girls participating in sports.*

P17 stated

*My father did not want me to join this area of study. He had the desire for me to join some other field, as my father does not like sport(s) and races. But on my insistence, he agreed to my joining this field of studies.*

Likewise, participants also disclosed the behaviors whereby others in social and academic domains sought to position them, and that they were often considered to be powerless. The majority of the young women who participated in this study were first-generation women in sports, and a few were first-generation women in higher education. These young women broke social barriers by selecting physical education and sports as a learning major for their master’s degrees. The decision to choose these major raised shared reflections among the young women, as they all required permission from their family to make this choice. For some, it was easy to decide due to having their parents’ support, but it was difficult for others, not primarily because of their parents, but due to other family members and society. Even if parents were supportive of the young women, they still had to deal with different patriarchal claims made by different people among their own families and communities.

Thoughts of P9 and P15’s were somehow similar they believed

*My family has no issue with my sports activities. However, my relatives object to my sports activities. They say that it is indecent for girls to do jumps and run races.*

Some of the statements these women and their parents were confronted with addressed issues such as different roles for women as a child and as a grownup, dressing, long-distance and traveling issues, laughing and shouting at women on public grounds, that the feminine body is inappropriate for sports, that sports are a male profession, and the male dominance in the faculty. Three interrelated themes were selected to explore the issues of women’s empowerment in sports for young women: resisting gender stereotypes, adjusting social beliefs, and rejecting social norms. These themes are used to reflect the victories and frustrations of our participants in implement them as strategies to achieve their goals.

### Resisting Gender Stereotypes: Developing Interest and Departure

The majority of the young women who participated in this research grew up in small cities where they had played on the streets in their childhood with other male and female friends from their neighborhoods. They were engaged in regional childhood sport(s) that were famous among children in Pakistan like other countries, such as hopscotch, rope skipping, barf pani (ice and water), hide and seek, oonch neech (up and down), pitho garam (seven stones), and paper games. As P8 shared about her childhood:

*I have loved playing sport(s) since my childhood. It was so much fun playing sport(s) with my friends and cousins. We played inside our homes, on roofs, inside rooms, and outside.*

P12 said

*I developed my interest in sports during my early years of life. I was very good at physical activities (running, jumping, throwing) because most of my childhood sport(s) included running, jumping, and throwing.*

Similar to P8 and P12, other young women shared similar memories of their childhood, and how active and happy they were in their childhood. However, they experienced a change in their daily life activities at home and school as they grew up. At home, social norms that required grownup women to stay indoors after school became dominant. While at school, certain notions of the “intelligent student” were dominant, referring to those who focused more on books and got good grades in tests. As P7 mentioned:

*When girls are children, they are permitted to play whatever they want. But when they grow up, they are asked to leave sport(s).*

P5 said

*I was active in physical activities when I was a child. I stopped playing sports from grade 6 due to several factors*… *lack of time due to school studies, homework at home, and social and family pressure. I started playing again in college during my bachelor’s degree.*

P11 believed that

*With age, society (…including neighbors, community, relatives, and family) has made it a social norm for girls to stay home and not play sports*… *especially outside.*

Similarly, P9 said

*I was very active in sporting activities at primary school. But later, I left sports because I was told that sports are not for women, and there is no career for women in sports. I occasionally practiced at home in my free time.*

Therefore, the opportunities for young women to play sports were minimized as they grew up. Like P11 and P9, participants identified that sports and physical activities were not suitable for women because the profession is not considered feminine in Pakistani society. Participants’ reflections highlighted cultural norms that consider women to be fragile commodities inappropriate for being subjected to hardship and laborious jobs (Shabib-ul-Hasan and Mustafa, 2014). Additionally, these norms include traditional expectations that women behave in a “ladylike” fashion (Wilde, 2007), which means wearing dresses, maintaining a beautiful and delicate body, being moral and pure, and raising children when they are grown adults (Wilde, 2007). Therefore, very little attention is paid to women’s physical activities in schools and at home. Furthermore, these reflections also support the notion that physical activity is not only for a professional career, but is important for a healthy and active body (Bird et al., 2013; Eime et al., 2013).

### Dealing With Dominant Discourses: Give and Take

To achieve change in families and communities, participants consistently perceived that a give and take strategy was necessary with respect to conforming to the dominant discourses. The “give and take” strategy was regarded as the dominant structure of knowledge and beliefs that encourages and allows a particular set of practices while discouraging and restricting the values of other practices. Participants often used this strategy by calculating the apparent costs and benefits of agreeing to social norms. However, dealing with dominant discourses by agreeing to social norms in terms of giving in order to achieve social and individual benefits is never simple. Participants regularly felt a high degree of pressure to follow or incorporate the major social norms. Additionally, the knowledge and beliefs presented as dominant discourses were not fixed, transparent, or gender neutral. This implies that participating in sports was not accessible to all on equal terms. Participants frequently discussed the uncomfortable and strange experiences of adapting to social norms and behaviors, and remained torn between conflicting social and ideological pressures. Therefore, participants often had to adapt to or assimilate social and family rules in order to achieve their own personal goals. As P1 said:

*In order to play sports and continue my studies, I had to understand the rules of my family and society. I was good at sports, but there was huge social pressure to leave sports because I am a woman. Even though my parents were supportive, I was not allowed to study in higher secondary school because many relatives were against women going for higher education and women playing sports due to relatives and community. But I did not give up; I studied at home, appeared at* the *exams as a private candidate, and passed them with good grades. These two years gave me the strength to fight for my rights. In these two years, I kept encouraging myself and saying to my parents that I would go to college if I passed the higher secondary exams.*

P1 built her position by completing her higher secondary education, which is necessary to enter higher education, even though she had to miss 2 years of her life at higher secondary school. She preserved firm control over her desire to play sports and achieve progress in her studies and career in sports. She observed great pressure from her relatives and community for her to stop playing sports and stop her studies. This is highly challenging for young women in families and communities with certain patriarchal beliefs. However, it appeared to be implicitly agreed among the participants that this was the only potential way for young women to deal with certain situations dominated by masculine norms and values. This strategy was considered necessary to make changes within a severely gendered system.

Additionally, as reflected by P1, social and cultural beliefs and values are a determining factor for women to respond and react in their life. Such position can also be beneficial for women as these experiences can help women re-evaluate their norms and values (Drewe, 2003; Bailey, 2006). Like AsmaP1, AsiaP2 shared a similar situation to that of P1, as her uncle and aunt were against her playing sports. However, in response to her constant efforts to talk to her uncle and aunt about sports, they put certain conditions on her playing sports:

*Sometimes, it is necessary to understand the situation and follow give and take. My uncle and aunt were against my participation in sports. After finishing my bachelor’s degree, I told them again that I wanted to select sports and physical education for my master’s degree. They agreed on one condition*… *that I would have to wait for one year. They put this condition because their daughter was studying in her second year, and she would start university next year. I think they thought it would be safe for us to go to university together.*

P2’s experience of showing the flexibility of waiting for one year to achieve her goals reflects the strong motivation to maintain her position in a predominantly patriarchal society. However, in this situation, it could be argued which rules can be flexible and to what extent, as well as to what degree women can accept the belief that women are incapable of working in male-dominated settings in the context of this flexible system with patriarchal principles. While P2 showed flexibility in accepting the condition of waiting for one year, her struggle in dealing with the dominant discourse has not finished yet. Having gained admission, she is required to go and return from university with her cousin. However, there are significant time differences between their classes, as well as the schedules for sports training and sports competitions; she often tries to negotiate with her teachers and trainers regarding the possibility of leaving early. Some of her teachers are supportive and understand her situation, but it is difficult for her to skip training and sports competitions. This situation is similar to that of many other participants who had agreed to co-operate with many social norms. Among many others, not playing in public places and not playing sports with men were major requirements that these young women had accepted in order to be able to play sports inside the campus. P3 said

…*I cannot go to public playgrounds, like many other girls, because boys mostly occupy public playgrounds, and my parents do not like it if we play in playgrounds. It is fine for me because I can play inside the campus.*

Similar to P3, P15 said

…*there are no separate playgrounds for girls in communities or universities. I often played in the park with my other female friends when I was in school, but many members of society complained to my father about our playing on public grounds. My father asked me to stop playing in the park, but I could play inside my school. I still follow this*… *Now I play inside the university but do not play in public playgrounds.*

In addition to the strategies for facing challenges at home and in society, participants revealed several challenges within their current physical education program and sport sciences. Even though we were not focusing on recording participants’ experiences of learning sports and physical education in their current program, participants frequently shared their personal and collective experiences of playing and learning sports in a male-dominated academic field. As discussed earlier, all full-time faculty in the department of sports and physical education were males. These reflections were mixed with respect to the participants’ expectations from the program, the opportunities, the difficulties of playing sports with other male students, and the difficulties of training with their male teachers. As P15 shared her personal experience,

*This program is very good, and all teachers are very supportive, but there is still a (communicative) gap between us*…*Even though I am very outspoken, I cannot share my things (women’s things) with teachers.*

Similar to P15, P17 shared a similar experience of playing sports with males and selecting it as a profession:

*Before enrolling in this program, I knew there were no female teachers in our department, but I prepared myself for this. However, it was not as expected it would be*… *on my first outdoor sports training, I had a (menstrual) period, I was in big pain, but I could not tell my teacher about my situation. It was a very painful experience, because it is impossible to share such things with others, especially males.*

### Rejecting Social Norms

A common theme surrounding women playing sports is that the women are resisting commonly believed notions of inferiority in sports and physical activities. Sports participation helps women realize that they are free from patriarchal demarcations and control and develop a sense of self-respect for their physical abilities (Eime et al., 2013). Many participants showed firm resistance by developing coping strategies when they felt marginalized. These strategies included rejection and refutation of the ideas imposed on them without their own choice, and which they regarded to be unimportant, strange, discriminatory, or even offensive. As P9 said:

*Sports and physical activities are sport(s), but they are completely disciplined. I get the awareness of the outside world through playing sports*…*I come to know how to present myself in front of this world as a player*…*I can feel I am powerful, not only physically, but also mentally*…

P9’s reflection demonstrates that sports and physical activities provide mental and physical strength to women, which is necessary to live a healthy life and face patriarchal behaviors. Similar to this, P13 shared her experience:

*My family was not against my sports participation, but they were against studying this subject as an academic major. But I told them I would choose this major only. Some of my relatives said it is indecent for girls to do jumps and run races. I told them, women are similar to men, and we can do everything that men can do.*

Similar to this, P3 said

…*our society does not like girls dressing like boys, and people do not like girls wearing tracksuits and short trousers to play sports. I have been criticized for wearing sports trousers. I rejected these notions because they are socially constructed ideas.*

These reflections from P13 and P3 show resistance to people’s patriarchal beliefs within families and communities. Both young women desired to build careers in sports, which was rare in their families and communities. Additionally, these young women suggested that girls should go out and break the barriers of the limited spaces available outside for girls to play and practice. This would create a sense of strength among women and create awareness that women are equal community citizens. P14 shared similar thoughts that women are equal citizens by law, and that there is no restriction on women playing in public playgrounds by law. However, there is a lack of awareness and a sense of danger among women that limits their chances of going out and playing in public spaces. She said

…*playing sports is good for me because it helped me overcome my shyness and develop confidence. I had to resist some social norms if I wanted to play sports. I opposed decisions on my sports participation, and I challenged many social norms that people believed against women’s participation in sports. Sports have made my behavior positive and constructive.*

Similar to these young women, P8 shared her experience of resistance from another perspective.

…*sports have taught me to fight and keep fighting till I win the game. Despite staying at home and earning money by teaching, which many of my former classmates are doing, I decided to join this program. Many people often ask me why I selected sports for my master’s degree? I ask them why they didn’t?*

### Raising an Empowered Generation: Important Elements

#### Parents and Family

In addition to norms suggesting different roles for women in society, the major source of inspiration that encouraged most of these young women to maintain their personal interest in playing sports was their parents, along with other family members. There was unconditional support from parents, siblings, cousins, and other relatives for the majority of these young women to participate in sports and physical activities. This is consistent with previous studies discussing the significant role of parents in providing the various opportunities and resources for their children that are required to cultivate their children’s sport development (Keegan et al., 2014; Knight and Holt, 2014).

As P16 said:

*My parents were very supportive in playing sports. They always supported me in playing sports. Even though I did not get many chances to play in my school years, they supported me when I told them I would choose physical education for my master’s studies.*

P2 reported

*My parents, especially my mom, was very supportive of my choices in my life. My house is far from the university, and we have evening competitions. My mom arranged a pick-and-drop service for me. Many of my relatives do not like it when I play sports or go back home late, but my mom always motivates me to do well in sports and does not pay attention to what people say.*

P8 said

*My parents and my three elder brothers supported me a lot. My brothers were very active in sports in their college years. One of my brothers is the principal of the primary school. He always encouraged me to play sports and play sports well. He always tells me I have to make our family proud of my sports success.*

Despite these amazing stories, some participants noticed that several family members were against their participation in sports. In addition, these young women had to sacrifice some of their individual choices, especially during their school years. However, as these young women grew up, they encouraged themselves to overcome the barriers and ignore the voices aiming to reduce their chances of achieving their goals. P4 lives far from the university campus, and she arranged a pick-and-drop service for herself. However, she always faced criticism from people in her neighborhood and her brother, who was against her going out alone and participating in sports. She said

*Pick and drop was a major issue for me, like many other girls around here. I had to go to playgrounds for practice, classes, and competitions at different times, sometimes early in the morning and sometimes in the evening. I arranged a pick-and-drop service for myself, but my brother is unwilling for me to travel alone. Even though he knows that I go alone, he still would not give me pick and drop.*

P4’s reflection provides a great example of how she supports herself to overcome the traveling problems and faces the pressure from her brother regarding her traveling alone. Her parents and other family members, including her female cousins, encouraged her to play sports despite her brother. Her female cousin, who was active in sports in higher secondary school, had participated in many regional badminton competitions. However, unfortunately, she left sports after finishing higher secondary school due to social and family issues and never participated in any competitions during college and university. P4’s cousin always encouraged her to pursue her career in sports if she was good at sports. Thus, as P4 said, the fact that no one can stop her from playing sport(s) acts as a symbol of liberation for women in society (Hamilton, 2012). Nevertheless, P2 faced a similar situation, but more complicated than P4. P2 had been an active participant in sports, but the unexpected death of her father when she was in high school changed the whole path of life for her. After her father’s death, her mother decided to move into her uncle’s (her mother’s brother) home together with her other siblings. Her mother and other siblings were always supportive of her participation in sports. However, her uncle was against women playing sports and did not permit her to compete in any competitions. She explained her situation as follows:

*I have been good at sports since my childhood, my parents always supported me, and I always wanted to participate in national competitions and make my parents proud. But my father’s sudden death changed everything for me*…*my dreams, plans, aspirations*… *all changed. It was difficult for my mom to raise all of the children alone, so she moved to my uncle’s (my mom’s brother’s) home. I had chances to apply for provincial interschool sport(s), but my uncle refused. In my higher secondary school, I convinced my mom, uncle and aunt to allow me once, only once, to participate in the trial for national sport(s) selection. They allowed me one week before the trials, and I was the only girl selected from among 40 girls throughout the district. But, my uncle and aunt did not give their permission to go to Islamabad to participate in a sports competition.*

P2’s story reflects the fact that her family may have sense that their social respectability would be endangered if she traveled alone to participate in sports. This reflects the position of women in Pakistani patriarchal society, where traveling can never be done easily by women, compared to men, and previous studies (see, e.g., Shabib-ul-Hasan and Mustafa, 2014; Fazal et al., 2019) have constantly reported the issues faced by women traveling in Pakistani society.

#### Women as Role Models

Women role models in sports and physical activities are considered essential for the inclusion of women in the sports field (Biskup and Pfister, 1999). These women role models have smoothed the way for generations of female players all over the world, struggling to overcome physical, social, financial, and political hurdles. Women role models help to increase the involvement of girls in sports activities (Spaaij, 2009). Their role and character, constantly produced and presented by the mass media, build dreams for people and make the world more energetic and interesting. They provide the necessary components to guide individuals with respect to the attitudes and appropriate ways of behaving during the socialization process. Similar to this, several participants mentioned that different athletes, especially women from Pakistan and other Islamic countries, had affected them by offering courage and hope. P2 said

…*I am impressed by the life of ‘Maria Toorapkai’. Whenever I face troubles in lif…the difficulties I had faced in the past…and the struggles I face at present, I always encourage myself by saying, ‘I must do it as Maria did it.’*

P2’s reflection on the meaningful importance of Maria Toorapkai in her life provides significant evidence that women’s struggle for empowerment has a great impact on other women in society, as well as future generations. Maria Toorapkai lived her childhood disguised as a “boy” and played sports with boys in her hometown, North Waziristan, previously controlled by the Taliban. Her participation in sports disguised as a boy, winning competitions by beating boys, playing sports by wearing man-like dresses, and her representation of Pakistan in international sports are elements that not only gave her a great reputation in Pakistan, but put her life in danger as well (see, e.g., Toorpakai and Holstein, 2017). However, her story of following her dreams, pursuing sports, and defying death threats has transformed her into a true inspiration for other women in Pakistan, as in the case of P2. Similarly, other participants also shared the effects of role models in their life for becoming change agents

P11 said

*I am inspired by Sania Mirza. She is not only an excellent tennis player, but also a great inspiration for young women like me.*

P14 said

*I get inspiration from all women who play at the national and international level, from all countries. I often watch women’s sports on TV, and I wish we could have our own (Pakistani) women’s team in every sport, just like cricket.*

In addition to national and international stars, many participants mentioned the names of people who were around them and had helped them at every step to become change agents. These included family members, teachers, friends, and self-motivation.

## Conclusion and Implications

This study explored the experiences of young women regarding their participation in sports and physical activities. It investigated tasks aiming to empower women in specific cultural and social contexts in the region in Pakistan in which women are least empowered by using feminism in sports theory. For this purpose, 18 young women undertaking a master’s in physical activities and sports were enrolled in this study, and shared their lived experiences of sports participation. These young women, who we refer to as change agents, were the first generation of women in their families to have selected physical activities and sports as learning disciplines, and in some cases were the first generation among their families to enroll in higher education. In examining these young women’s regular practices as change agents, the study highlighted how they confronted the visible gender inequalities that affected them and employed different strategies to empower themselves to change this patriarchal structure. Focusing on the interviews, the young women reported having to confront gender stereotypes within their families and communities. A second practice reported by participants was the strategy of compromising on a few issues in order to achieve their goals and complete their mission of participating in sports and physical activities. The majority of the participants reported avoiding playing sports in public spaces because of social attitudes toward women and the limited availability of women-only spaces, similar to previous studies. However, a key factor in their success as change agents was the support of their parents in achieving their targets, and the effect of the lived experiences of other female athletes similar to these young women. These findings are similar to those reported in previous studies in the Pakistani context as well as other world regions (see, e.g., Dagkas et al., 2011; Laar et al., 2019b).

The young women who participated in this study had practiced further strategies for change. They resisted, negotiated, incorporated, or completely rejected social norms and their associated discourses in order to accomplish their priorities and desires. Through resistance to a series of gender stereotypes during different stages of adolescence, they aimed to construct substitute methods of subjectivity as an alternative to the reality of women’s behavior being controlled. However, the participants employed different resistance strategies, with some incorporating some of the dominant discourses through a give and take strategy, which was articulated as being an uncomfortable situation by several participants. The situations confronted by these young women reflected the typical attitude of society, which disapproves of women’s freedom, possibly because it is assumed to threaten male dominance. The findings are similar to this reported in previous studies on women in Pakistan (Fazal et al., 2019), personal resilience and desire to excel were reported as empowering factors for Pakistani women.

The lived experiences of the young women to become change agents, as explained above, have several implications for internal and external policy domains at the local and national levels. Any new policies or development models aimed at empowering women to play sports and participate in physical activities against patriarchal norms and creating resistance toward traditional concepts of physically active women should consider the lived experiences of young women and involve the multiple forms of agencies that these young women have developed. Policymakers need to consider the attitudes of family and other people in communities toward or against women playing sports and participating in physical activities, especially in public places. Therefore, strategies and structures should be formulated to provide equal rights for young women to play sports in male-occupied public places. Policies and development models supporting the representation of women athletes will help to direct the chances of emergence of different hierarchies resulting from women’s participation in sports and the complexities of their everyday lives. Additionally, university administrations must understand the complex realities of Pakistani patriarchal society and strive to provide better facilities that encourage young women to play sports and learn physical activities. Apart from these, there is an urgent need to create awareness among people, societies, and schools of the importance and possible benefits of sports to women. Therefore, a sporting and physical activity framework with equal participation from women is essential to revive the sports culture (Laar et al., 2019a). These policies and frameworks can build a conducive environment that maximizes women’s involvement in sports and physical activities (Campbell, 1990; Yenilmez, 2021).

The research findings represented the personal experiences of participation in sports and physical activities of the 18 participants from different regions in the south of Punjab. A limitation of the current study is that the sample size may not represent women nationwide. Moreover, this research did not precisely focus on participants’ experience playing sports at a university with a male-only faculty. However, these in-depth interviews with the young women on their lived experiences of participation in sports and physical activities before enrolling in university also provide sufficient new knowledge and call for more future research on women’s participation in sports and physical activities, as well as identifying new change agents in different regions of Pakistan. This research further identifies that there is abundant space for both qualitative and quantitative research focusing on the impact of sports and physical activities on women’s empowerment and the experience of women working in sports and physical activities. In addition, future research focused on the individual and collective experiences of young women playing and learning sports and physical activities in specific academic majors would be of great value.

## Data Availability Statement

The raw data supporting the conclusions of this article will be made available by the authors, without undue reservation.

## Ethics Statement

The study was conducted according to the guidelines of the Declaration of Helsinki, and approved by the Institutional Review Board (or Ethics Committee) of Hubei Normal University. The patients/participants provided their written informed consent to participate in this study.

## Author Contributions

SP and RAL: conceptualization and writing—original draft preparation. SP: methodology. MAA: writing—review and editing. All authors have read and agreed to the published version of the manuscript.

## Conflict of Interest

The authors declare that the research was conducted in the absence of any commercial or financial relationships that could be construed as a potential conflict of interest.

## Publisher’s Note

All claims expressed in this article are solely those of the authors and do not necessarily represent those of their affiliated organizations, or those of the publisher, the editors and the reviewers. Any product that may be evaluated in this article, or claim that may be made by its manufacturer, is not guaranteed or endorsed by the publisher.
